# Resveratrol-Schiff Base Hybrid Compounds with Selective Antibacterial Activity: Synthesis, Biological Activity, and Computational Study

**DOI:** 10.3390/microorganisms10081483

**Published:** 2022-07-22

**Authors:** Rodrigo Sánchez-González, Patricio Leyton, Luis F. Aguilar, Mauricio Reyna-Jeldes, Claudio Coddou, Katy Díaz, Marco Mellado

**Affiliations:** 1 Instituto de Química, Facultad de Ciencias, Pontificia Universidad Católica de Valparaíso, Valparaíso 2373223, Chile; rodrigo.sanchez.g@pucv.cl (R.S.-G.); patricio.leyton@pucv.cl (P.L.); luis.aguilar@pucv.cl (L.F.A.); 2 Departamento de Ciencias Biomédicas, Facultad de Medicina, Universidad Católica del Norte, Coquimbo 1781421, Chile; mauricio.reyna@ucn.cl (M.R.-J.); ccoddou@ucn.cl (C.C.); 3 Millennium Nucleus for the Study of Pain (MiNuSPain), Santiago 8330025, Chile; 4 Núcleo para el Estudio del Cáncer a Nivel Básico, Aplicado y Clínico, Universidad Católica del Norte, Antofagasta 1270709, Chile; 5 Departamento de Química, Universidad Técnica Federico Santa María, Valparaíso 2390123, Chile; 6 Instituto de Investigación y Postgrado, Facultad de Ciencias de la Salud, Universidad Central de Chile, Santiago 8330507, Chile

**Keywords:** resveratrol, Schiff base, *Listeria monocytogenes*, selectivity, virtual screening

## Abstract

Nowadays, antimicrobial resistance is a serious concern associated with the reduced efficacy of traditional antibiotics and an increased health burden worldwide. In response to this challenge, the scientific community is developing a new generation of antibacterial molecules. Contributing to this effort, and inspired by the resveratrol structure, five new resveratrol-dimers (**9a**–**9e**) and one resveratrol-monomer (**10a**) were synthetized using 2,5-dibromo-1,4-diaminobenzene (**8**) as the core compound for Schiff base bridge conformation. These compounds were evaluated in vitro against pathogenic clinical isolates of *Pseudomonas aeruginosa*, *Staphylococcus aureus*, *Bacillus* sp., and *Listeria monocytogenes*. Antibacterial activity measurements of resveratrol-Schiff base derivatives (**9a**–**9e**) and their precursors (**4**–**8**) showed high selectivity against *Listeria monocytogenes*, being 2.5 and 13.7 times more potent than chloramphenicol, while resveratrol showed an EC_50_ > 320 µg/mL on the same model. Moreover, a prospective mechanism of action for these compounds against *L*. *monocytogenes* strains was proposed using molecular docking analysis, finding a plausible inhibition of internalin C (InlC), a surface protein relevant in bacteria–host interaction. These results would allow for the future development of new molecules for listeriosis treatment based on compound **8**.

## 1. Introduction

Antimicrobial resistance (AMR) has evolved into an urgent public health issue. This phenomenon is where pathogens use their genomic plasticity, adaptation potential, mutagenic rate, and gene transfer mechanisms to alter their cell morphology and processes [1]. An example of this phenomena is the antibacterial resistance acquired by human commensal microflora, which can use the aforementioned mechanisms to increase their virulence [2]. This bacterial adaptability severely compromises antibiotic efficacy and their clinical outcomes against infectious diseases [3,4]. In this sense, estimated mortality rates indicate that deaths associated with infectious diseases will surpass cancer-related demises (8.2 million) in 2050 [5]. Many of these deaths are linked with AMR, and pathogenic strains of *Pseudomonas aeruginosa*, *Staphylococcus aureus*, *Bacillus* sp., and *Listeria monocytogenes* are among the deadliest throughout the world [4,6,7,8,9,10]. Regarding *P. aeruginosa*, this Gram-negative pathogen is commonly associated with opportunistic nosocomial infections [7,11], especially in immunocompromised patients [11]. Conversely, *S. aureus* and *Bacillus* sp. are Gram-positive bacteria [8,9,10] commonly associated with episodes of food poisoning, minor skin infections (e.g., abscesses), and life-threatening diseases (e.g., pneumonia, meningitis, endocarditis, and sepsis) [12]. Lastly, *L. monocytogenes* is a Gram-positive, non-spore forming, facultatively anaerobic bacterium whose complex pathogenesis produces a rare but potentially serious infection called listeriosis [4,6]. Although most of the listeriosis cases can be considered as mild illnesses or even be unnoticed, they can progress to systemic listeriosis, which is associated with high mortality rates (20–30%) [13], even with early antibiotic treatment [4,6]. This life-threatening situation is more frequently observed in immunosuppressed patients, the elderly, and pregnant women [4].

**Figure 1:**
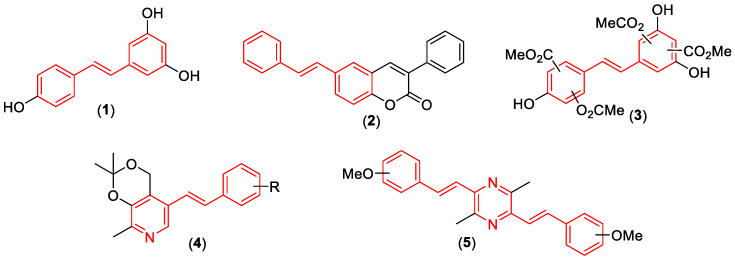
Resveratrol and hybrid resveratrol compounds with biological activity (Compounds **1**–**5**). The styrene core is highlighted in red.

In recent years, the scientific community has been exploring natural compounds in search for a plausible solution to this health concern, finding several bioactive compounds such as resveratrol (**1**). This molecule, normally found in fruits and vegetables, is a styrene-core compound (highlighted in red in Figure 1) [14] that has been associated with anticancer [15], antioxidant [16], anti-inflammatory [17], anti-neurodegenerative [18], and antibacterial effects [19]. These promising biological activities depict resveratrol as a valuable moiety to synthetize resveratrol-hybrids with improved effects (Figure 1), e.g., coumarin-resveratrol hybrids (**2**) with MAO-B inhibition activity [20]; aspirin-resveratrol derivatives (**3**) as anti-inflammatory agents [21]; ligustrazine-resveratrol compounds (**4**) with anti-ischemic effects [22]; pyridoxine-resveratrol hybrids (**5**) as MAO-B inhibitors [23], among others. Regarding antibiotic activity, researchers have found that Schiff base derivatives have potent antiproliferative effects against Gram-positive and Gram-negative bacteria, with previously reported activities against *S. aureus*, *P. aeruginosa*, *Streptococcus pyogenes*, *Escherichia coli*, and *L. monocytogenes* [24,25,26,27,28,29].

**Figure 2:**
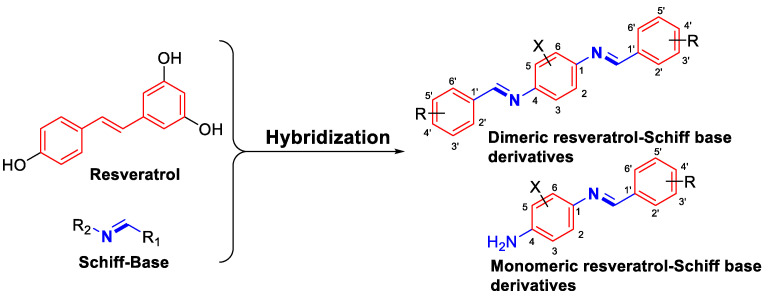
Hybridization strategy for resveratrol-Schiff base derivatives.

Considering the imperative need for novel antibiotics that could subvert AMR, and taking account of (1) the promising antimicrobial effects of resveratrol against Gram-positive and Gram-negative bacteria; (2) the bacteriostatic capacity of resveratrol on some bacterial strains; and (3) the demonstrated antibacterial activity of Schiff base derivatives, we performed an isosteric change in the styryl fragment, using different Schiff bases as structural bridges to obtain a new generation of prospective antibacterial agents (Figure 2). With this rationale, five novel resveratrol-Schiff base dimers (**9a**–**9e**) and one monomer (**10a**) were obtained, using four traditional synthetic steps with some modifications. For antibacterial activity assessment, intermediate compounds (**6**–**8**) and resveratrol-Schiff base derivatives (**9a**–**9e** and **10a**) were tested in vitro against pathogenic strains of *S. aureus*, *P. aeruginosa*, *Bacillus* sp., and *L. monocytogenes*. Finally, a structure-activity relationship (SAR) study was carried out to explain these effects, and a molecular docking study was performed to propose a potential mechanism of action for these compounds.

## 2. Materials and Methods

### 2.1. General

The melting point was measured using a Stuart Scientific Melting Point SMP3 apparatus (Staffordshire, UK). Infrared spectra were recorded using a Jasco FT-IR 4600 spectrometer (Tokyo, Japan). ^1^H-NMR (300 MHz) and ^13^C-NMR (75 MHz) were recorded on a Fourier 300 FT-NMR Spectrometer System (Berlin, Germany), using tetramethylsilane (TMS) as the internal standard. Chemical shifts were reported in δ (ppm downfield from the TMS resonance), and coupling constants (*J*) are given in Hz. GC-MS was carried out using a Shimadzu Europe GCMS-QP5050A spectrometer (Kyoto, Japan).

### 2.2. Chemistry

The following reagents were purchased from Sigma Aldrich-Merck and used without any prior treatment: 2,5-dibromoaniline (**6**, >98%), glacial acetic acid (>99%), acetic anhydride (>99%), tin(II)-chloride dihydrate (98%), benzaldehyde (99%), *p*-anisaldehyde (98%), 4-(trifluoromethyl)benzaldehyde (98%), *o*-hydroxybenzaldehyde (98%), 4-pyridinecarboxaldehyde (97%), and resveratrol (98%). The hydrochloric, nitric, and sulfuric acids were from LaboChem (Athens, Greece), and isopropanol, methanol, ethanol, hexane, ethyl acetate, and acetone were purchased from J.T. Baker (Radnor, PA, USA).

#### 2.2.1. Synthesis of *N*-(2,5-Dibromo-4-nitrophenyl)acetamide (**7**)

The first step to obtain this compound (**7**) is by performing the acetylation of 2,5-dibromoaniline (**6**). For this purpose, in a 250 mL bottom flask, compound **6** (5.00 g, 19.9 mmol) and 12.0 mL of acetic acid were mixed. Later, acetic anhydride (12.0 mL, 127 mmol) at 0 °C was added. This mixture was heated to 70 °C for 30 min and then cooled to room temperature. Exceeding amounts of acetic anhydride were discarded with 100 mL of distilled water. The obtained product, 2,5-dibromoacetanilide (**6**-Ac, 95% of yield), was a white solid which was filtered and washed with abundant water, and its identity was analyzed, obtaining the following parameters: Melting point (Mp) = 170–172 °C, FT-IR(KBr) ν 3282, 1664, 1522, 1389, 1280, 1036, 798 cm^−1^. These spectroscopic results are consistent with previous reports [30,31].

Afterwards, into a 250 mL bottom flask, compound **6**-Ac (5.44 g, 18.58 mmol) was mixed with H_2_SO_4_ (13 mL, 98% *w*/*w*) at −10°C. Subsequently, an H_2_SO_4_/HNO_3_ (1:1) solution was added dropwise (25 mL) to the previous mixture while holding the temperature at −10°C for 30 min. To terminate this reaction, cold distilled water was added (150 mL), obtaining a yellow solid. This powder was washed with distilled water and purified by re-crystallization in ethanol, obtaining compound **7** (81% of yield). The identification parameters for this compound were the following: Mp = 178–180 °C, FT-IR(KBr) ν 3295, 1672, 1505, 1346 cm^−1^. These measurements were consistent with previous reports [32].

#### 2.2.2. Synthesis of 2,5-Dibromobenzene-1,4-diamine (**8**)

In a 250 mL bottom flask, compound **7** (2.50 g, 7.40 mmol) was mixed with 15.0 mL of absolute ethanol. Next, a solution of SnCl_2_ × 2H_2_O (6.70 g, 29.7 mmol) and 37.0 mL of HCl (0.1 N) was added. This mixture was heated between 70–80 °C for 2 h and then cooled to room temperature. Afterwards, the exceeding HCl was neutralized using NaOH (50% *w*/*v*), forming a white solid as the product. This solid was filtered and washed with abundant distilled water, obtaining compound **8** (91% of yield) as the product. The obtained identification parameters were the following: Mp = 175°C, FT-IR(KBr) ν 3366, 3173, 1619 cm^−1^. The spectroscopic results are consistent with those previously reported [30,32].

#### 2.2.3. General Procedure for Schiff Bases Synthesis (**9**–**10**)

In a 100 mL bottom flask, 0.500 g of compound **8** (1.92 mmol) and 15.0 mL of EtOH were mixed, and 2.5 equivalents of aryl-aldehyde (4.80 mmol) were mixed. This reaction was stirred to reflux for 2 h. The solid formed was vacuum-filtered and washed with cold methanol. Finally, these solids were purified by re-crystallization, using different solvents depending on the obtained derivative: acetone (compound **9a**), ethyl acetate (compounds **9b**, **9c**, and **9e**), and isopropanol (compound **9d**). Compound **10a** was purified using column chromatography and eluted with ethyl acetate.

The obtained identification parameters for these derivatives were the following:

- (1*E*,1′*E*)-*N,N*′-(2,5-dibromo-1,4-phenylene)bis(1-(pyridin-4-yl)methanimine) (**9a**): Yellow solid (50% of yield). Mp: 272–274 °C, FT-IR(KBr): υ 3025, 2901, 1629, 1598 cm^−1^. ^1^H-NMR (300 MHz, CDCl_3_, δ, ppm): 8.82 (4H, dd, *J* = 4.47, 1.31 Hz, H7), 8.44 (2H, s, H4), 7.83 (4H, dd, *J* = 4.47 Hz, *J* = 1.59 Hz, H6), 7.41 (2H, s, H2). ^13^C-NMR (75 MHz, CDCl_3_, δ, ppm): 159.9 (C4), 150.8 (C7), 148.3 (C3), 141.9 (C5), 123.4 (C2), 122.5 (C6), 118.4 (C1). EI-MS *m/z*: 446 [M + 2]^+^, 444 [M]^+^, 442 [M − 2]^+^.
- (1*E*,1′*E*)-*N,N*′-(2,5-dibromo-1,4-phenylene)bis(1-phenylmethanimine) (**9b**): Pale yellow solid (82% of yield). Mp: 209–211 °C, FT-IR(KBr): υ 3072, 3053, 3019, 1625, 1575 cm^−1^. ^1^H-NMR (300 MHz, CDCl_3_, δ, ppm): 8.41 (2H, s, H4), 7.95 (4H, dd, *J* = 7.98, 2.33 Hz, H6), 7.52 (6H, m, H7 + H8), 7.35 (2H, s, H2). ^13^C-NMR (75 MHz, CDCl_3_, δ, ppm): 161.9 (C4),148.7 (C3), 135.7 (C5), 132.2 (C8), 129.4 (C6), 129.0 (C7), 123.5 (C2), 118.2(C1). EI-MS *m/z*: 444 [M + 2]^+^, 442 [M]^+^, 440 [M − 2]^+^.
- (1*E*,1′*E*)-*N,N*′-(2,5-dibromo-1,4-phenylene)bis(1-(4-methoxyphenyl)methanimine) (**9c**): Pale yellow solid (71% of yield). Mp: 220–223 °C, FT-IR(KBr): υ 3071, 2927, 2834, 1622, 1571 cm^−1^. ^1^H-NMR (300 MHz, CDCl_3_, δ, ppm): 8.33 (2H, s, H4), 7.92 (4H, d, *J* = 8.8 Hz, H6), 7.35 (2H, s, H2), 7.02 (4H, d, *J* = 8.8 Hz, H7), 3.88 (6H, s, H9). ^13^C-NMR (75 MHz, CDCl_3_, δ, ppm): 162.7 (C8), 160.8 (C4), 148.4 (C3), 130.9 (C6), 128.6 (C5), 123.2 (C2),118.1 (C1), 114.3 (C7), 55.5 (C9). EI-MS *m/z*: 504 [M + 2]^+^, 502 [M]^+^, 500 [M − 2]^+^.
- (1*E*,1′*E*)-*N,N*′-(2,5-dibromo-1,4-phenylene)bis(1-(4(trifluoromethyl)phenyl)methanimine) (**9d**): Pale yellow solid (80% of yield). Mp: 185–187 °C, FT-IR (KBr): υ 2923, 1627, 1578 cm^−1^. ^1^H-NMR (300 MHz, CDCl_3_, δ, ppm): 8.48 (2H, s, H4), 8.12 (4H, d, *J* = 8.20 Hz, H6), 7.77 (4H, d, *J* = 8.20 Hz, H7), 7.40 (2H, s, H2). ^13^C-NMR (75 MHz, CDCl_3_, δ, ppm): 160.2 (C4), 148.4 (C3), 138.5 (C5), 133.7 (_2_J^13^C-^19^F = 32.8 Hz, C8), 129.4 (C6), 125.9 (_3_J^13^C-^19^F = 3.73 Hz, C7), 125.6 (C9), 123.3 (C2), 118.3 (C1). EI-MS *m/z*: 580 [M + 2]^+^, 578 [M]^+^, 576 [M − 2]^+^.
- 2,2′-((1*E*,1′*E*)-((2,5-dibromo-1,4-phenylene)bis(azaneylylidene))bis(methaneylylidene))diphenol (**9e**): Orange solid (73% of yield). Mp: 280–282°C, FT-IR(KBr): υ 3447, 1624, 1609, 1570 cm^−1^. ^1^H-NMR (300 MHz, CDCl_3_, δ, ppm): 12.86 (2H, s, H11), 8.66 (2H, s, H4), 7.61 (2H, s, H2), 7.46 (4H, m, H8 + H10), 7.00 (4H, m, H7 + H9). ^13^C-NMR (75 MHz, CDCl_3_, δ, ppm): 165.6 (C6), 160.9 (C4), 145.5 (C3), 134.7 (C5), 133.6 (C10), 123.9 (C8), 120.2 (C2), 119.9 (C9), 119.5 (C7), 117.2 (C1). EI-MS *m/z*: 476 [M + 2]^+^, 474 [M]^+^, 472 [M − 2]^+^.
- (*E*)-2,5-dibromo-4-((pyridin-4-ylmethylene)amino)aniline (**10a**): Yellow solid (9% of yield). Mp: 146–147 °C, FT-IR(KBr): υ 3466, 3306, 1630, 1570 cm^−1^. ^1^H-NMR (300 MHz, (Acetone-d_6_, δ, ppm): 8.75 (2H, dd, *J* = 6.1, 1.58 Hz, H7), 8.66 (1H, s, H4), 7.88 (2H, dd, *J* = 6.1 Hz, 1.58 Hz, H6), 7.57 (1H, s, H2), 7.27 (1H, s, H9), 5.34 (2H, s, H11). ^13^C-NMR (75 MHz, CDCl_3_, δ, ppm): 156.2 (C4), 150.6 (C7), 144.1 (C10), 142.6 (C5), 140.0 (C3), 122.3 (C6), 122.2 (C2), 120.7 (C8), 119.0 (C9), 108.2 (C1). EI-MS *m/z*: 357 [M + 2]^+^, 355 [M]^+^, 353 [M − 2]^+^.

### 2.3. Bacterial Strains

The bacterial strains were clinical isolates that belong to the Biological Tests Laboratory collection (Chemistry Department, Universidad Técnica Federico Santa María). These isolates correspond to *P. aeruginosa*, *S. aureus*, *Bacillus* sp., and *L. monocytogenes*. The strains were cultured and stored in Mueller–Hinton Broth (MHB, Difco, Detroit, MI, USA) at 37°C and Mueller–Hinton agar (Difco, Detroit, MI, USA), respectively.

### 2.4. In Vitro Antibacterial Activity Assays

Resveratrol, resveratrol-Schiff base derivatives, and their precursors (**9a**–**e**, **10a**, and **6**–**8**, respectively) were dissolved in dimethyl sulfoxide (DMSO), and their stock solutions were prepared using sterile distilled water as a solvent, obtaining a final DMSO concentration of less than 1% in each well so as not to affect bacterial growth. Percentage Growth Inhibition (PGI) was calculated for each pathogenic bacterium against all the evaluated compounds using a modified serial dilution method [33], testing all the compounds in a concentration range between 5.0 and 320 μg/mL (5.0, 10, 20, 40, 80, 160, and 320 μg/mL). An equal volume (1.5 μL) of bacterial suspension containing 10^6^ CFU/mL was inoculated into sterile 96-well microplates (considering 200 μL as the final volume) and incubated aerobically at 37 °C for 24 h on a shaker at 120 rpm. PGI was calculated according to OD600 readings obtained from a Thermo Scientific Multiskan GO 96-well plate spectrophotometer (Waltham, MA, USA). Chloramphenicol (CALBIOCHEM; San Diego, CA, USA and Ottawa, ON, Canada) was used as the positive control, using the same concentration gradient for the bacterial strains, and 1% DMSO with an inoculum condition was used as the negative control. An additional condition consisting of 1% DMSO without bacteria was used to subtract background OD600 values. Each compound concentration was assayed in triplicate, and each informed value represents the mean ± SD of two independent experiments. Antibacterial activity was categorized into four different levels from most to least active according to the percentage of inhibition (% I) at 320 µg/mL: very highly active (++++, 80–100% I), highly active (+++, 60–80% I), moderately active (++, 40–60% I), slightly active (+, 20–40% I), and inactive compound (−, 0–20% I). The half maximal effective concentration (EC_50_) was obtained for each compound by fitting their PGI (%) and concentrations in a dose-response equation [34,35]. Fit analysis was performed using Origin 8.0 software.

### 2.5. Statistical Analysis

The data were reported as mean values ± standard deviation (SD). One-way ANOVA and post hoc HSD Tukey tests were used, considering a confidence level of 0.95. Statistical significance was calculated, making comparisons between the antibacterial activities for each synthetized compound and those obtained for chloramphenicol. Statistical analyses were performed using the Statistica 7.0 software (StatSoft, Inc., Tulsa, OK, USA).

### 2.6. Molecular Docking Analysis

Molecular docking experiments were carried out according to previous reports from our research group [36,37]. Crystalline structures from positive regulatory factor A (PrfA, PDB ID: 5LRR) [38], penicillin-binding protein 4 (PBPs4, PDB ID: 3ZG8) [39], internalin A (PDB ID: 1O6V) [40], internalin B (PDB ID: 2WQU) [41], and internalin C (PDB ID: 1XEU) [42] were downloaded from the Protein Data Bank (PDB). The 3D-structures of each active compound (**6**, **7**, **8**, **9a**, **9b**, **9c**, and **10a**) were generated with the ChemDraw software (Perkin Elmer, Waltham, MA, USA). Using the AutoDock 4.2 software [43], rotatable bonds of each ligand were assigned, polar hydrogen atoms were added, and water molecules were removed from the PDB files. Docking analysis of each ligand was performed using the AutoDock Vina script [44] with a grid box of 20·20·20 Å (8000 Å^3^) and a 0.375 Å space centered in the active site for each protein. This region was defined using the top-ten ranked docking poses that were saved for each docking run. The molecular docking results were processed using Pymol [45] to identify each ligand–protein interaction.

## 3. Results and Discussion

### 3.1. Chemistry

**Scheme 1:**
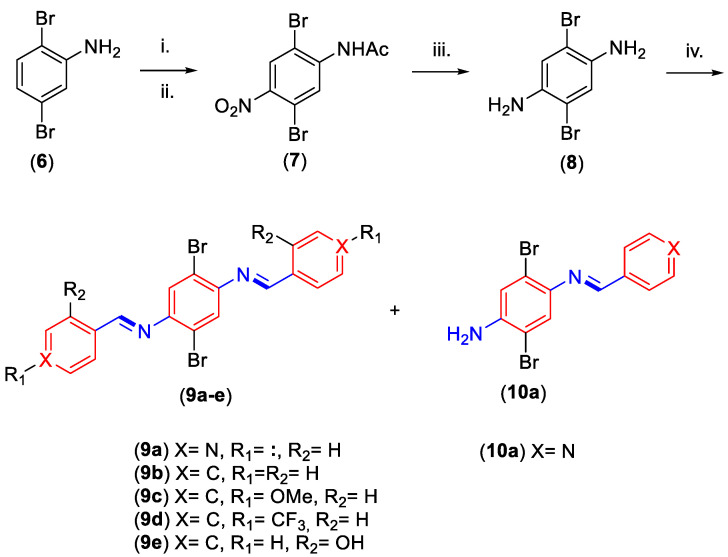
Synthetic steps to obtain the resveratrol-Schiff base derivatives **9**–**10**. General conditions: (**i**) Ac_2_O, H^+^, 70–80 °C, 30 min, 95%; (**ii**) HNO_3_/H_2_SO_4_, −10 °C, 30 min, 81%; (**iii**) SnCl_2_ × 2H_2_O, HCl, EtOH, reflux, 2 h, 91%; (**iv**) 2.5 equivalents of aromatic aldehyde, EtOH, reflux, 2 h, 50–84%.

In order to obtain the resveratrol-Schiff base dimers and monomers (**9**–**10**, respectively), it is first is necessary to obtain the 1,4-diaminobenzene core (**8**, see Scheme 1). To do this, we started with 2,5-dibromoaniline (**6**) and performed an acetylation reaction using traditional procedures in acidic media, obtaining a 95% yield and confirming the desired product by the presence of a signal at 1667 cm^−1^ (C=O stretch) [30,46]. After this, the amide of compound **6** was selectively nitrated in *para*-position from the amide substituent, achieving an 81% yield and ensuring chemoselectivity by the steric hindrance of the -NHAc group, which blocks the *ortho*-oriented nitration, according to a previous report [46]. The obtained *N*-(2,5-dibromo-4-nitrophenyl)acetamide (**7**) identity was confirmed by referential signals at 1505 and 1346 cm^−1^ (-NO_2_ stretching) and at 3296 cm^−1^ and 1673 cm^−1^ (amide fragment) [32,47].

The nitro fragment of compound **7** was reduced to an amino group in order to synthetize 2,5-dibromobenzene-1,4-diamine (**8**). To do this, compound **7** was treated with SnCl_2_ × 2H_2_O in acidic media to obtain compound **8** with a 91% yield. We confirmed this reduction reaction by checking the absence of signals at 1505 and 1346 cm^−1^ (-NO_2_ stretching) [46]. Once the diamine derivative **8** was synthetized, we went on to obtain the dimeric and monomeric resveratrol-Schiff base derivatives **9** and **10**, respectively. The resveratrol-Schiff base dimers (**9a**–**e**) were obtained by the condensation of compound **8** with 2.5 equivalents of aromatic aldehyde under reflux conditions, reaching moderate to high yields (50–82%). The compounds were identified by complementary spectroscopic techniques (IR, NMR, and MS). In the IR spectra, all compounds showed signals around ~3040 cm^−1^ (=C-H stretching) and a peak at ~1625 cm^−1^ (C=N stretching), which is characteristic of the imine groups. The ^1^H-NMR spectra at downfield showed a singlet signal (δ~8.54 ppm) corresponding to the HC=N group hydrogen. In the ^13^C-NMR spectra, all the compounds showed a low field signal (δ~160 ppm), representative of the C=N carbon. The EI-MS spectra of all compounds (**9a**–**e**) showed three *m/z* signals, each one of them corresponding to the [M + 2]^+^, [M]^+^, and [M − 2]^+^ ions, characteristic of the isotopic mark of Br-79 and Br-81 [48].

Resveratrol-Schiff base monomer (**10a**) synthesis is possible exclusively when compound **8** is condensed with pyridine-4-carbaldehyde. Due to the excess of aromatic aldehyde and its low reactivity in this situation, the monomeric resveratrol-Schiff base derivative can only be obtained as a secondary product, achieving a 9% yield for compound **10a**. Spectroscopic information confirmed that the desired compound (**10a**) showed IR spectral signals at 3466 and 3306 cm^−1^ (-NH_2_ stretching) and at 1630 cm^−1^ (C=N stretching). The ^1^H-NMR spectra showed a broad singlet signal at δ = 5.35 ppm, corresponding to the two hydrogens of the amino group (-NH_2_). Additionally, compound **10a** showed ^13^C-NMR and EI-MS spectra with similar characteristics to those obtained for dimeric resveratrol-Schiff base derivatives.

### 3.2. In Vitro Antibacterial Activity

Resveratrol, synthetic intermediates, and resveratrol-Schiff base derivatives (**6–8**; **9a**–**c** and **10a**) were assessed as antibacterial agents using the microdilution method against pathogenic clinical isolates of Gram-negative (*P. aeruginosa*) and Gram-positive (*S. aureus*, *Bacillus* sp., and *L. monocytogenes*) bacteria. Initially, we evaluated the antibacterial activity of all these compounds, except for the water-insoluble derivatives **9d** and **9e**, at 320 µg/mL on each bacterial culture (the results are summarized in Table 1). These results show that all the assessed compounds have different antibacterial activity profiles against the evaluated pathogens. These variations can be explained through the pleiotropic effects of resveratrol [19], which can reduce bacterial proliferation by interacting with multiple molecular targets. These effects could be also observed, to a greater or lesser extent, in our resveratrol-Schiff base derivatives. In descending order, these compounds have antibacterial activity against *L. monocytogenes* > *P. aeruginosa* > *Bacillus* sp. > *S. aureus*. The calculated EC_50_ values against each pathogen are detailed in Table 1.

When we analyzed the effects of the evaluated compounds against *P. aeruginosa*, we observed a high-activity profile (++++), similar to the one observed for the positive control, with the exception of compounds **9b** and **10a**, which showed moderate activity (++), similar to resveratrol, and were 8.7-fold less active than the resveratrol-Schiff base derivative **9a**, which exhibited the best activity profile against this pathogen. Regarding the calculated EC_50_ values, compounds **6** and **8** (EC_50_ = 18.72 ± 0.97 and 21.49 ± 1.50 µg/mL, respectively) appeared as the most potent derivatives against *P. aeruginosa*, but they were between 1.7- and 3.1-fold less active than chloramphenicol (EC_50_ < 5.0 µg/mL, Table 1). Regarding the resveratrol-Schiff base derivatives, compounds with a pyridine fragment, such as compound **9a**, showed an EC_50_ = 26.04 ± 1.18 µg/mL, but another compound with the same moiety (compound **10a**) exhibited reduced antibacterial activity against this pathogen (EC_50_ > 320 µg/mL). These effects could be attributed to the lipophilicity and symmetry of compounds **9a** and **10a**, where the symmetrical nature of the dimeric resveratrol-Schiff base derivative **9a** is associated with a greater lipophilicity (CLogP = 3.10) than that of the asymmetric monomeric derivative **10a** (CLogP = 2.06). This feature facilitates drug transit from the extracellular medium to the highly lipidic plasmatic membrane of Gram-negative bacteria [49,50].

When the antibacterial activity against Gram-positive bacteria (*S. aureus*, *L. monocytogenes*, and *Bacillus* sp.) was analyzed, we found different activity profiles for each one of these pathogenic strains. For example, the dimeric resveratrol-Schiff base derivative **9c** has an -OMe substituent and shows proper activity against *L. monocytogenes* (EC_50_ = 10.07 ± 1.31 µg/mL) and *Bacillus* sp. (EC_50_ < 5 µg/mL) but reduced antibacterial effects on *S. aureus* (EC_50_ > 320 µg/mL). Conversely, compound **9a** is one of the most active compounds against *L. monocytogenes* (EC_50_ = 1.43 ± 0.60 µg/mL) and is also one of the least potent agents against *S. aureus* (EC_50_ > 320 µg/mL) and *Bacillus* sp. (inactive). Regarding the latter effects, the results show, in agreement with a previous report, an increased activity for dimeric resveratrol-Schiff base derivatives (symmetric compounds) in comparison to their monomeric counterpart (asymmetric molecule) [3]. Despite these observations, all the evaluated compounds showed weaker antibacterial effects on *S. aureus* than the positive control chloramphenicol (EC_50_ < 5 µg/mL), resveratrol being the representative with the second highest activity against this strain (EC_50_ = 152.21± 0.03 µg/mL). Regarding *Bacillus* sp., precursor molecule **8** showed no activity, resveratrol exhibited a mild effect (EC_50_ > 320 µg/mL), and the resveratrol-Schiff base derivatives **9b**, **9c**, and **10a** were more active (EC_50_ < 5 µg/mL) than chloramphenicol (EC_50_ = 18.20 ± 0.69 µg/mL). Regarding the effects on *L. monocytogenes*, most of the evaluated compounds showed high antibacterial activity at 320 µg/mL, inhibiting bacterial growth by 80–100% (++++). These compounds presented similar (e.g., **9c**) or higher (e.g., **7**, **8**, **9a**, **9b**, and **10a**) EC_50_ values than chloramphenicol (see Table 1); however, the natural compound resveratrol had low activity against this pathogenic strain (20–40% inhibition at 320 µg/mL, Table 1). Regarding synthetic intermediates, the results show that compound **6** has the lowest activity of all the assessed compounds (EC_50_ = 24.29 ± 1.02 µg/mL), while their nitro-derivative (**7**) increased its antibacterial activity by 7.9-fold (EC_50_ = 3.07 ± 0.38 µg/mL), this effect being more potent than the one observed for the positive control chloramphenicol (EC_50_ = 10.33 ± 1.61 µg/mL, *p* < 0.05) and consistent with previous antibacterial activity assessments of nitro-aromatic derivatives [51]. Furthermore, di-amine compound **8** shows an increased inhibition of *L. monocytogenes* growth when compared to compound **6** (*p* < 0.05, Table 1) and similar activity compared to the nitro-derivative **7** (*p* > 0.05, Table 1). Moreover, dimeric resveratrol-Schiff base derivatives (**9a**–**9c**) showed antibacterial activity similar to that of its precursor (**8**, *p* > 0.05). As previously mentioned, these effects could be attributed to the lipophilicity of each compound but also to Schiff base derivatives hydrolysis [52] and the plausible oxidation of compound **8** to a molecule similar to cyclohexa-2,5-diene-1,4-diimine [53].

Our results are consistent with those reported by other authors regarding the diverse effects of resveratrol against different strains of Gram-positive or Gram-negative bacteria under similar experimental conditions [19,54]. For example, some resveratrol derivatives inhibited the growth of Gram-negative bacteria at concentrations higher than 100 µg/mL, but Gram-positive bacteria showed a higher sensibility to this agent. This phenomenon can be explained by the poor penetration capacity of resveratrol through the outer membrane of Gram-negative bacteria or by its efflux by bacterial pump systems. Additionally, because resveratrol can inhibit ATP synthase in different bacterial species, resveratrol susceptibility profiles could be explained by the specific metabolic requirements of each pathogenic strain [55,56]. Finally, these results portray resveratrol-Schiff base derivatives, both the dimeric and monomeric representatives, as compounds with higher antibacterial activity than resveratrol, achieving similar effects by using lower concentrations of these agents.

### 3.3. Molecular Docking

With the aim to elucidate a potential antibacterial mechanism of action behind the high-potency effects against *L. monocytogenes* for our synthetized compounds (**6**–**10**), we analyzed the Protein Data Bank (PDB) database to evaluate possible interactions between the resveratrol-Schiff base derivatives and proteins from the *Listeria* genus. As these compounds do not have previous reports of their mechanism of action, we performed a virtual screening technique according to prior studies in order to find a plausible molecular target [36]. In line with this, we analyzed proteins related with *Listeria* development and pathogenesis in the PBD database [38,57], finding that the positive regulatory factor A (PrfA, PDB ID: 5LRR) [38], the penicillin-binding protein 4 (PBPs4, PDB ID: 3ZG8) [39], and the internalin forms A, B, and C (PDB IDs: 1O6V, 2WQU, and 1XEU, respectively) are potential molecular targets [40,41,42].

The affinity energies obtained in the molecular docking analysis performed against the active site of the potential molecular targets of resveratrol-Schiff base derivatives (**9**–**10**), their synthetic precursors (**6**–**8**), and resveratrol are summarized in Table 2 and Figures S1–S4. In this table, resveratrol-Schiff base derivatives and their precursors (including the native ligand) showed negative docking scores, revealing that these molecules could have a spontaneous interaction with these proteins. In the case of PrfA (PDB ID: 5LRR), despite the negative values obtained for the analyzed compounds, its native ligand exhibited a lower score than compounds **6**, **8**, and **10a** (ΔG= −6.1 kcal/mol, Table 2), meaning that these compounds cannot displace the native ligand–PrfA interaction, excluding PrfA as a potential molecular target. This same trend is seen for PBPs4 (PDB ID: 3ZG8), internalin A (PDB ID: 1O6V), and internalin B (PDB ID: 2WQU), so these targets were also discarded from the analysis. Finally, when we observed the docking scores for internalin C (PDB ID: 1XEU), we found that our synthetized compounds showed better affinity energy values than the native ligand (ΔG < −3.6 kcal/mol). This information reveals that these resveratrol-Schiff base derivatives and their precursors could displace the native ligand from its interaction with internalin C and exert an antibacterial effect. Indeed, when we performed a linear relationship between experimental EC_50_ and the affinity energies obtained for internalin C, we obtained acceptable values for Pearson’s correlation coefficient (r1XUE = 0.630). On the other hand, resveratrol and the native ligand of internalin C showed identical affinity energies (ΔG = −3.6 kcal/mol), meaning that resveratrol cannot displace the native ligand from its interaction. This potential internalin C inhibition by the resveratrol-Schiff base derivatives and their synthetic intermediaries can be associated with a blockade of bacterial invasion and an adhesion to human epithelial cells on *L. monocytogenes* [58]. This prospective interaction is in accordance with the previous bacteriostatic effects reported for resveratrol [19].

**Figure 3:**
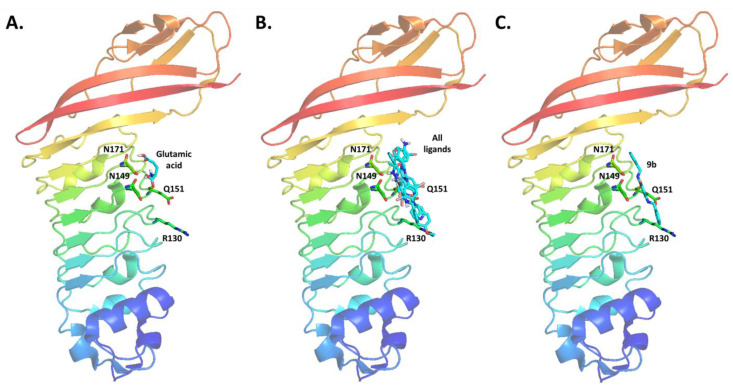
Molecular docking results for internalin C (PDB ID: 1XEU). (**A**). 3D-structure overview of internalin C with its native ligand. (**B**). 3D overview of internalin C and resveratrol-Schiff base derivatives and their precursors. (**C**). Detailed polar and van der Waals interactions between compound **9b** and the internalin C aminoacidic residues.

Regarding the internalin C protein structure, the active site is located in the concave face of its three-dimensional (3D) structure [42] (Figure 3A). When our molecular docking results for the resveratrol-Schiff base derivatives and their synthetic precursors (compounds **6**–**8**, **9a**–**c**, and **10a**) were contrasted against the structure of internalin C, a favorable spatial orientation near the N171 and N149 residues was observed (Figure 3B). These results confirm a potential glutamic acid displacement induced by the evaluated synthetic compounds. When our results for the most active compound (**9b**, Figure 3C) were analyzed, we observed polar and van der Waals interactions with the N171 and N149 residues of internalin C, which are similar to those observed for the native ligand. These van der Waals interactions between compound **9b** and the active site of internalin C are stabilized with additional interactions with residues Q151 and R130 (red dashed lines, Figure 3C). Comparing the other resveratrol-Schiff base derivatives with the interactions observed for compound **9b**, we observed that the addition of an electron donor group (-OMe, compound **9c**) in the *para*-position of the benzene ring increases its negative density. This effect can explain the affinity energy decrease by an electronic repulsion with the R130 and N171 residues. Moreover, the synthetic precursors and monomeric resveratrol-Schiff base derivative (e.g., **6**, **7**, **8**, and **10a**) showed lower affinity energies than the dimeric derivatives **9a**–**c**. This effect could be related with the interaction with N171 and R130 residues, located at both extremes of the structure of these symmetric compounds. Internalin C inhibition could be related to the bacteriostatic effect of these molecules because this protein is associated with the adhesion and invasion of *L. monocytogenes* to epithelial cells [58]. Conversely, resveratrol has a perpendicular orientation towards internalin C, forming only a hydrogen bond with Q151 (see Figure S6), which is located far from the active site of this protein (N171 and N149). These results are in accordance with the reduced antibacterial activity observed for resveratrol against *L. monocytogenes* (Table 1).

An interesting trend was observed when the high-affinity energies of resveratrol-Schiff base derivatives with a better performance than the native ligand were analyzed against the active sites of the remaining target proteins (Table 2). This analysis revealed that antibacterial compounds **9a**–**9c** could also act as potential inhibitors against PrfA, PBPs4, internalin A, or internalin B.

## 4. Conclusions

Six novel resveratrol-Schiff base derivatives—five symmetric (**9a**–**e**) representatives and one asymmetric (**10a**) representative—were synthetized using a four-step chemical procedure, obtaining global yields between 6 and 57% (**9a**-35%, **9b**-57%, **9c**-50%, **9d**-56%, **9e**-51%, and **10a**-6%) and verifying their chemical identity by traditional spectroscopic techniques.

Symmetrical resveratrol-Schiff base derivatives showed reduced antibacterial activity against *S. aureus*, while on *P. aeruginosa*, some resveratrol-Schiff base derivatives and their precursors exhibited better activity than resveratrol but were less potent than the positive control chloramphenicol. However, when we analyzed the antibacterial effects against *Bacillus* sp., an intermediate effect was observed for these synthetic compounds, and resveratrol did not show any activity on this pathogenic strain. Interestingly, all resveratrol-Schiff base derivatives exhibited potent antibacterial activity against *L. monocytogenes*, showing similar (e.g., **9c**) or even higher (e.g., **9a**–**9b**, *p* < 0.05) effects than chloramphenicol. These antibacterial activities against *L. monocytogenes* could be explained by the lipophilicity increase observed for symmetric resveratrol-Schiff base derivatives, a feature that improves the entrance of active compounds through bacterial membranes.

Finally, after performing a molecular docking virtual screening, internalin C was identified as a plausible target. This prospective mechanism of action is in accordance with previous reports of the bacteriostatic effects of resveratrol. Despite these approaches, further experiments must be performed in order to confirm this prospective mechanism. With all this information, resveratrol-Schiff base derivatives appear as a promising alternative for the development of antibacterial compounds against *L. monocytogenes*.

## Figures and Tables

**Table 1:** Antibacterial activity levels and calculated EC_50_ values of synthesized compounds against pathogenic bacterial strains.

Comp	CLogP ^†^	*P. aeruginosa*		*S. aureus*		*L. monocytogenes*		*Bacillus* sp.	
		Activity	EC_50_ ^a^	Activity	EC_50_ ^a^	Activity	EC_50_ ^a^	Activity	EC_50_ ^a^
**Res ^c^**	2.83	+++	226.97 ± 0.07	+++	152.21 ± 0.03	+	>320	+	>320
**6**	3.07	++++	18.72 ± 0.97	++	305 ± 0.65	++++	24.29 ± 1.02	++	>320
**7**	2.97	++++	43.20 ± 0.99	+	>320	++++	3.07± 0.38 *	−	−
**8**	1.96	++++	21.49 ± 1.50	++	>320	++++	1.00± 0.32 *	−	−
**9a**	3.10	++++	26.04 ± 1.18	+	>320	++++	1.43 ± 0.60 *	−	−
**9b**	5.58	++	>320	+	>320	++++	0.75 ± 0.25 *	++++	ODSC
**9c**	6.27	++++	40.0 ± 0.95	+	>320	++++	10.07 ± 1.31	++++	ODSC
**9d**	7.35	In ^b^	In ^b^	In ^b^	In ^b^	In ^b^	In ^b^	In ^b^	In ^b^
**9e**	6.05	In ^b^	In ^b^	In ^b^	In ^b^	In ^b^	In ^b^	In ^b^	In ^b^
**10a**	2.06	++	>320	++	>320	++++	5.02 ± 1.02 *	++++	ODSC
**C^+^**	1.28	++++	ODSC	++++	ODSC	++++	10.33 ± 1.61	++++	18.20 ± 0.69 *

^†^ CLogP= Lipophilicity index calculated by ChemDraw. ^a^ EC_50_ = Half maximal effective concentration (µg/mL). ^b^ In = Insoluble in water. ^c^ Res = Resveratrol. C^+^ = Chloramphenicol. Antibacterial activity at 320 µg/mL: ++++ = very highly active (80–100% inhibition), +++ = highly active (60–80% inhibition), ++ = moderately active (40–60% inhibition), + = slightly active (20–40% inhibition), and − = inactive compound (0–20% inhibition). ODSC= Out of Dose-Response Curve, EC_50_ < 5.0 µg/mL. *n* = number of true replicates for each experiment, *n* = 3. * *p* < 0.05.

**Table 2:** Docking scores of resveratrol-Schiff base derivatives, their precursors, and resveratrol on different target proteins related to Listeria genus bacteria development and pathogenesis.

Compound	Calculated Affinity Energy (kcal/mol)				
	5LRR	3ZG8	1O6V	2WQU	1XEU
**Res ^a^**	−6.6	−6.1	−4.3	−4.6	−3.6
**6**	−4.9	−4.3	−3.7	−3.5	−3.7
**7**	−6.2	−5.8	−3.9	−4.4	−4.5
**8**	−5.1	−4.6	−3.9	−4.1	−3.9
**9a**	−7.6	−7.1	−5.0	−4.9	−5.1
**9b**	−7.5	−6.8	−5.2	−5.1	−5.1
**9c**	−7.3	−6.3	−5.5	−4.6	−4.4
**10a**	−5.9	−6.1	−4.5	−4.6	−4.8
**NL ^b^**	−6.1	−5.8	−4.3	−4.1	−3.6

^a^ Res = Resveratrol. ^b^ NL = Native ligand. Protein Data Bank IDs: 5LRR = PrfA, 3ZG8 = PBPs4, 1O6V = Internalin A, 2WQU = Internalin B, and 1XEU = Internalin C.

## Data Availability

Not applicable.

## Supplementary Materials

The following supporting information can be downloaded at: https://www.mdpi.com/article/10.3390/microorganisms10081483/s1, Spectras S1–S18: FT-IR, ^1^H-NMR, and ^13^C-NMR of symmetric imines (**9a**–**9e**) and asymmetric imine (**10a**); Figures S1–S6: Molecular docking results. Spectra S1: FT-IR of compound **9a**; Spectra S2: ^1^H-NMR of compound **9a**; Spectra S3: ^13^C-NMR of compound **9a**; Spectra S5: FT-IR of compound **9b**; Spectra S6: ^1^H-NMR of compound **9b**; Spectra S7: ^13^C-NMR of compound **9b**; Spectra S8: FT-IR of compound **9c**; Spectra S9: ^1^H-NMR of compound **9c**; Spectra S10: ^13^C-NMR of compound **9c**; Spectra S11: FT-IR of compound **9d**; Spectra S12: ^1^H-NMR of compound **9d**; Spectra S13: ^13^C-NMR of compound **9d**; Spectra S14: FT-IR of compound **9e**; Spectra S15: ^1^H-NMR of compound **9e**; Spectra S15: ^13^C-NMR of compound **9e**; Spectra S16: FT-IR of compound **10a**; Spectra S17: ^1^H-NMR of compound **10a**; Spectra S18: ^13^C-NMR of compound **10a**; Figure S1: Molecular docking results for active derivatives on positive regulatory factor A (PrfA, PDB iD: ILRR); Figure S2: Molecular docking results for active derivatives on penicillin-binding protein 4 (PBPs4, PDB iD: 3ZG8); Figure S3: Molecular docking results for active derivatives on internalin A (PDB iD: 1O6V); Figure S4: Molecular docking results for active derivatives on internalin B (PDB iD: 2WQU); Figure S5: Molecular docking results for active derivatives on internalin C (PDB iD: 1XEU); Figure S6: Molecular docking results for compound **9b** and resveratrol on internalin C (PDB iD: 1XEU).
