# Supplementary material for: Resveratrol-Schiff Base Hybrid Compounds with Selective Antibacterial Activity: Synthesis, Biological Activity, and Computational Study
Source: Microorganisms. 2022 Jul 22;10(8):1483. doi: 10.3390/microorganisms10081483 (PMC9330556; doi:10.3390/microorganisms10081483)
Supplement: Supplementary file 1 [file microorganisms-10-01483-s001.zip › microorganisms-1789674-supplementary.pdf]

SUPPLEMENTARY MATERIAL

Communication

Resveratrol-Schiff base hybrid compounds with selective antibacterial activity: Synthesis, biological activity, and computational study

Rodrigo Sánchez-González [1], Patricio Leyton [1], Luis F. Aguilar [1], Mauricio Reyna-Jeldes [2-4], Claudio Coddou [2-4], Katy Díaz [5,*], Marco Mellado [6,*]

[1] Instituto de Química, Facultad de Ciencias, Pontificia Universidad Católica de Valparaíso, Valparaíso 2373223, Chile; rodrigo.sanchez.g@pucv.cl (R.S-G.); patricio.leyton@pucv.cl (P.L.)
[2] Departamento de Ciencias Biomédicas, Facultad de Medicina, Universidad Católica Del Norte, Coquimbo 1781421, Chile; mauricio.reyna@ucn.cl (M.R-J.); ccoddou@ucn.cl (C.C.)
[3] Millennium Nucleus for the Study of Pain (MiNuSPain), Santiago 8330025, Chile; mauricio.reyna@ucn.cl (M.R-J.); ccoddou@ucn.cl (C.C.)
[4] Núcleo para el Estudio del Cáncer a Nivel Básico, Aplicado y Clínico, Universidad Católica del Norte, Antofagasta 1270709, Chile; mauricio.reyna@ucn.cl (M.R-J.); ccoddou@ucn.cl (C.C.)
[5] Departamento de Química, Universidad Técnica Federico Santa María, Valparaíso 2390123, Chile; katy.diaz@usm.cl (K.D.)
[6] Instituto de Investigación y Postgrado, Facultad de Ciencias de la Salud, Universidad Central de Chile, Santiago 8330507, Chile; marco.mellado@ucentral.cl (M.M.)
* Correspondence: katy.diaz@usm.cl (K.D.); marco.mellado@ucentral.cl (M.M.)

Table of Content

Spectra S1-S18: FT-IR, ^{1}H-NMR and ^{13}C-NMR sprectra for symmetric (9a-9e) and asymmetric imines (10a)........ 2

Figures S1-S6: Molecular docking results 14

Spectra S1-S18: FT-IR, ^{1}H-NMR and ^{13}C-NMR spectra for symmetric (9a-9e) and asymmetric (10a) imines

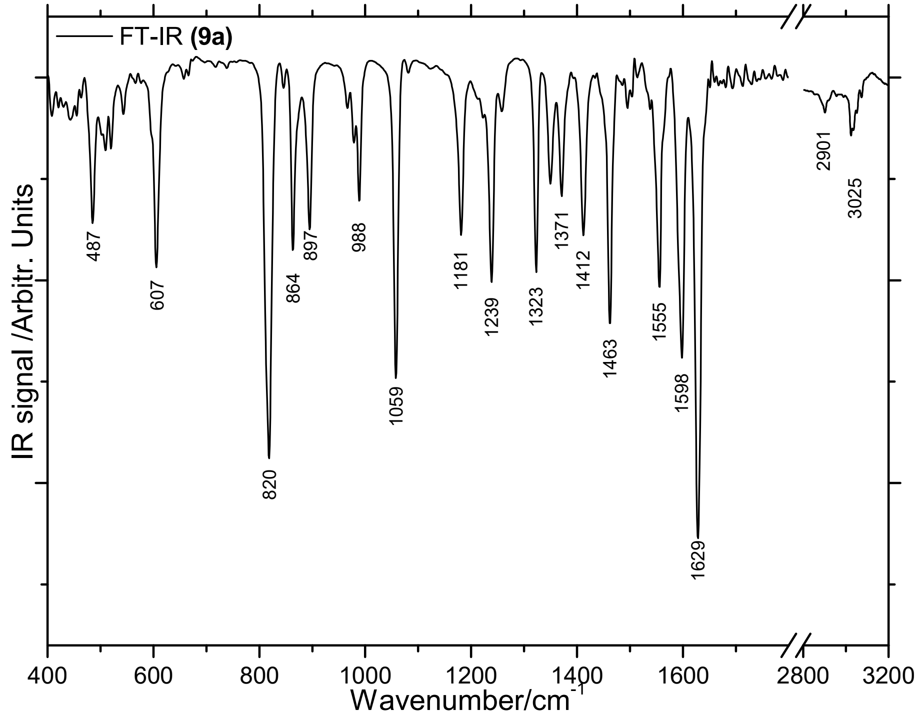

Spectra S1: FT-IR of compound **9a**

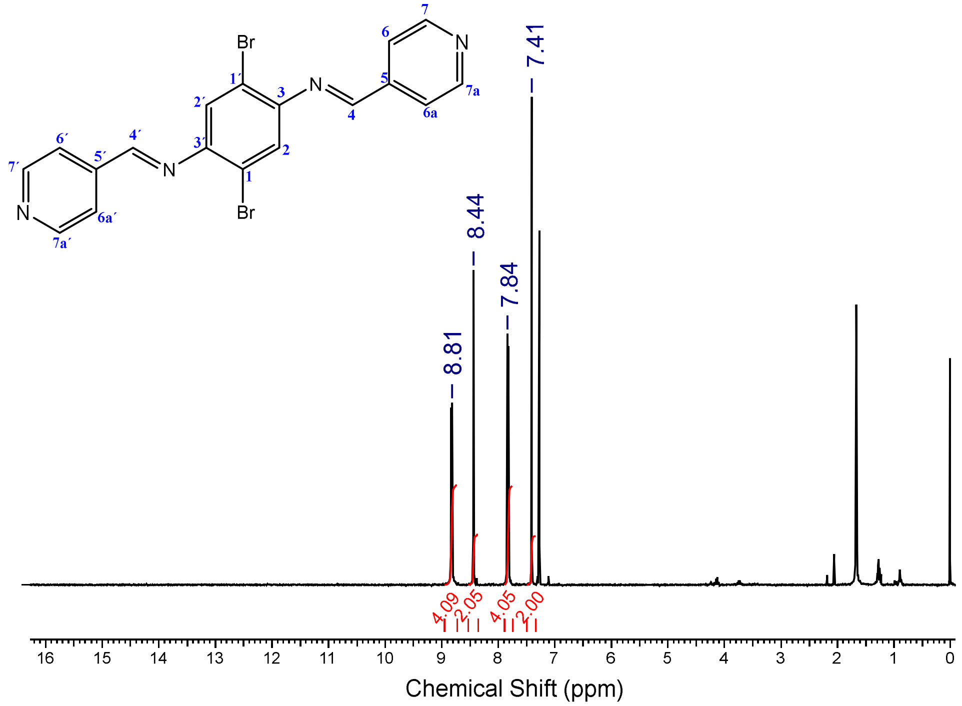

Spectra S2: ^{1}H-NMR of compound **9a**

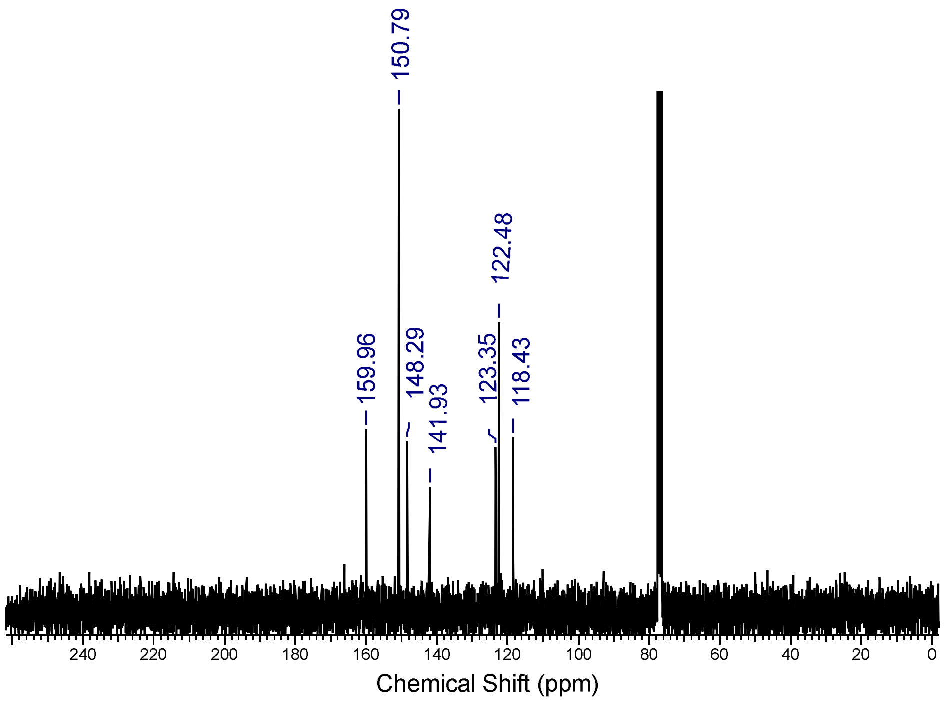

Spectra S3: ^{13}C-NMR of compound **9a**

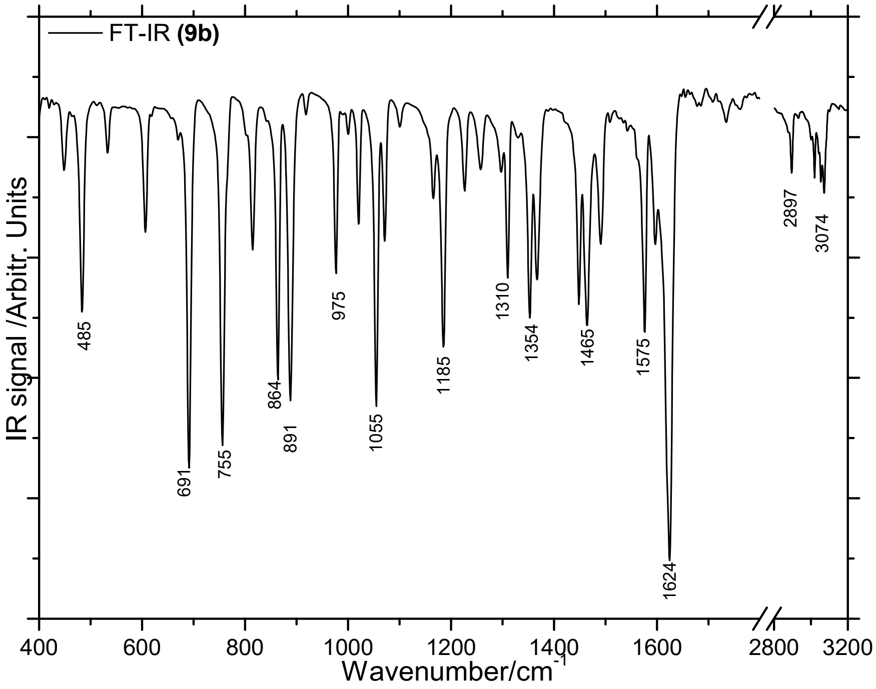

Spectra S4: FT-IR of compound **9b**

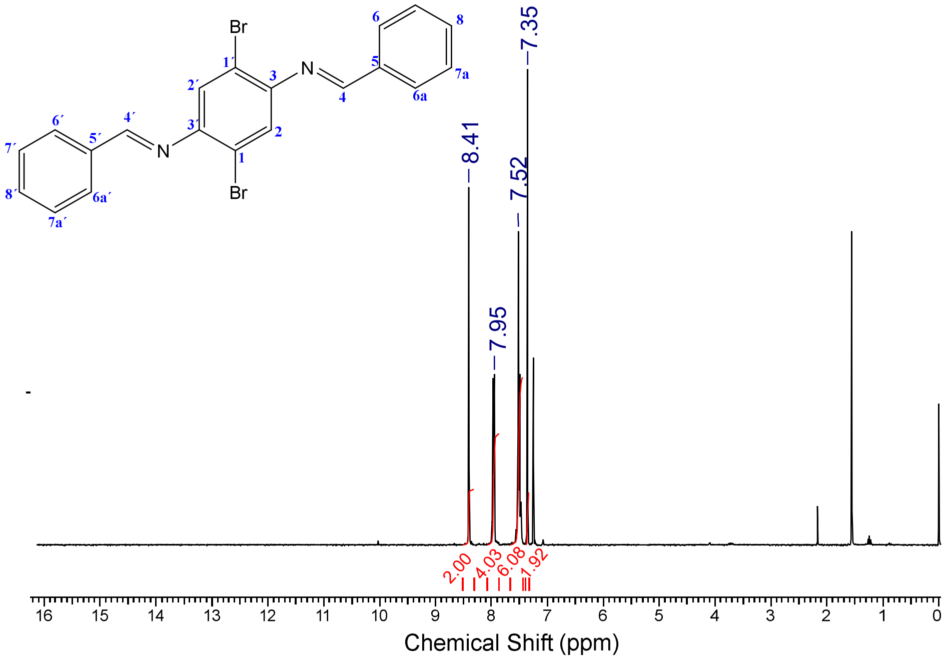

Spectra S5: ^{1}H-NMR of compound **9b**

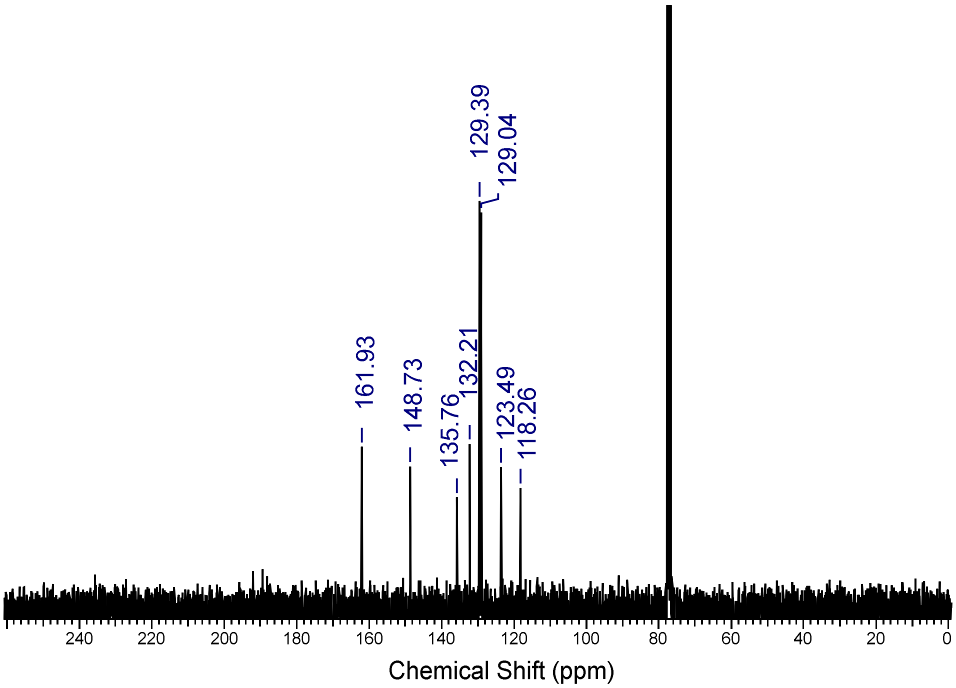

Spectra S6: ^{13}C-NMR of compound **9b**

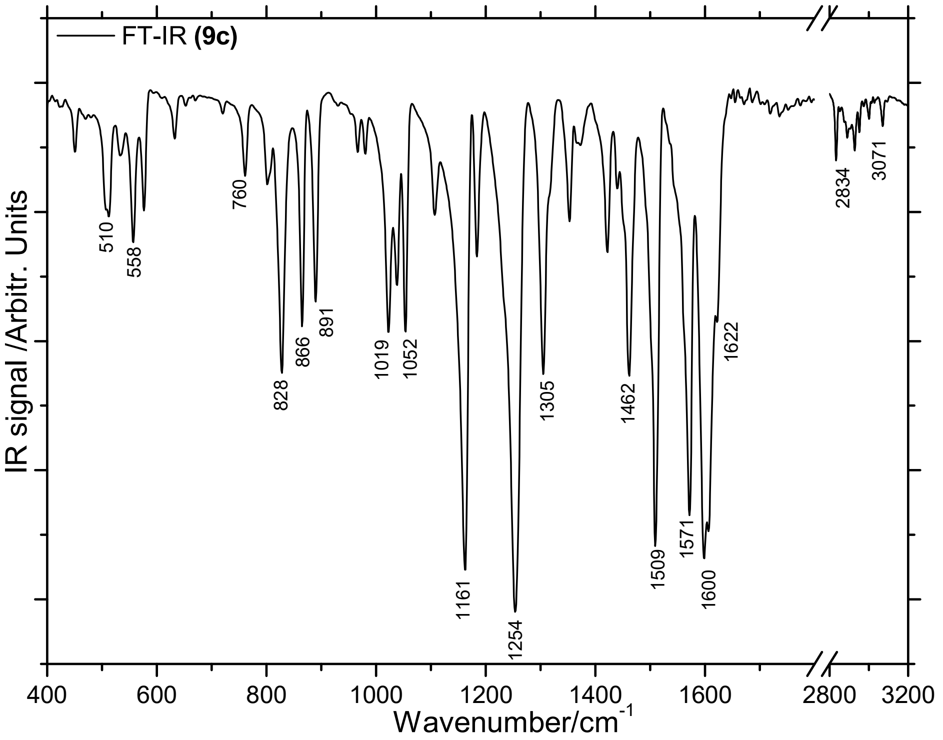

Spectra S7: FT-IR of compound **9c**

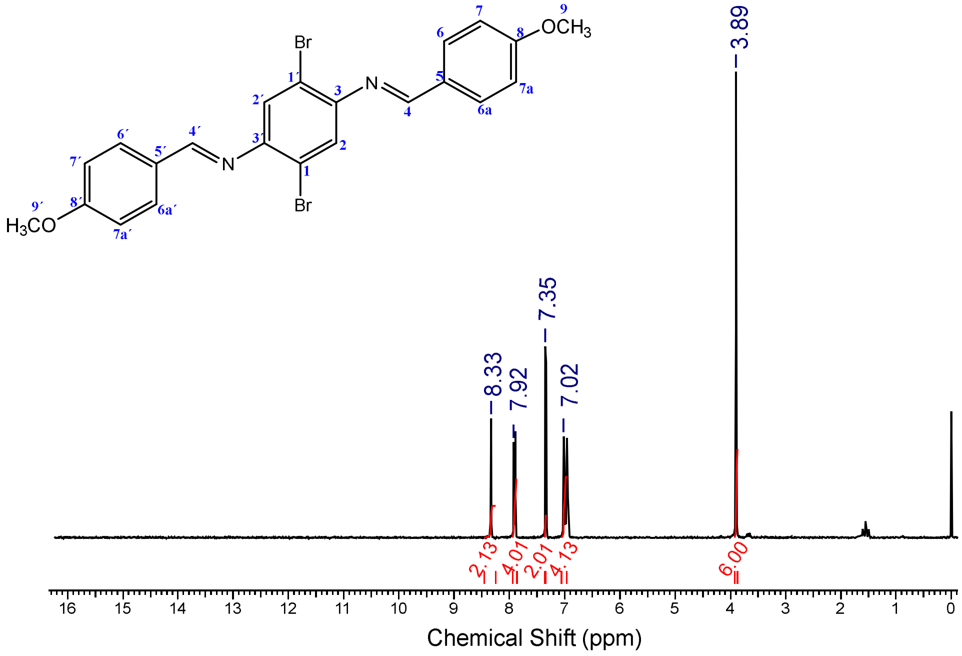

Spectra S8: ^{1}H-NMR of compound **9c**

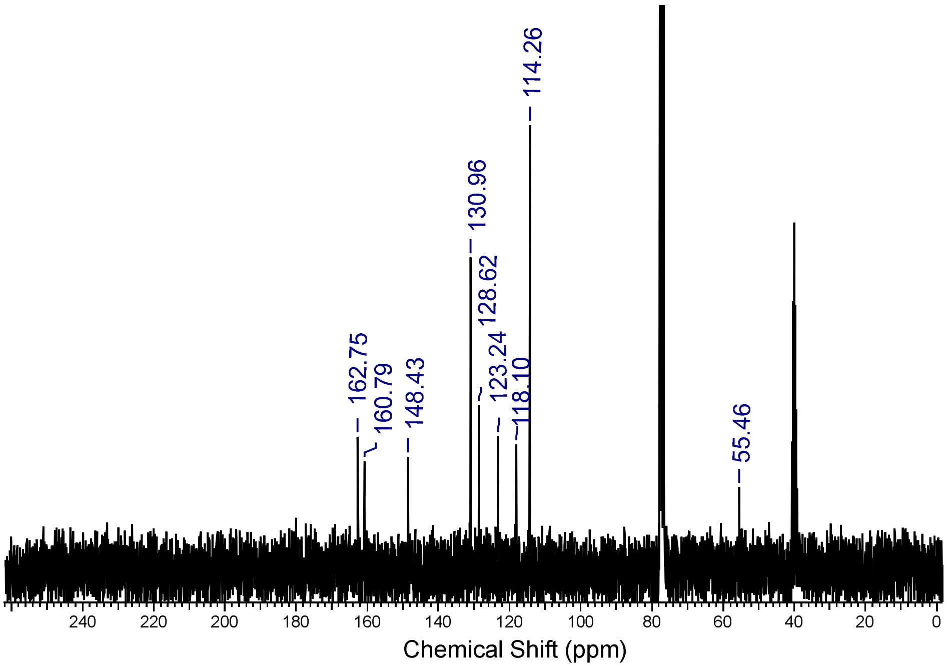

Spectra S9: ^{13}C-NMR of compound **9c**

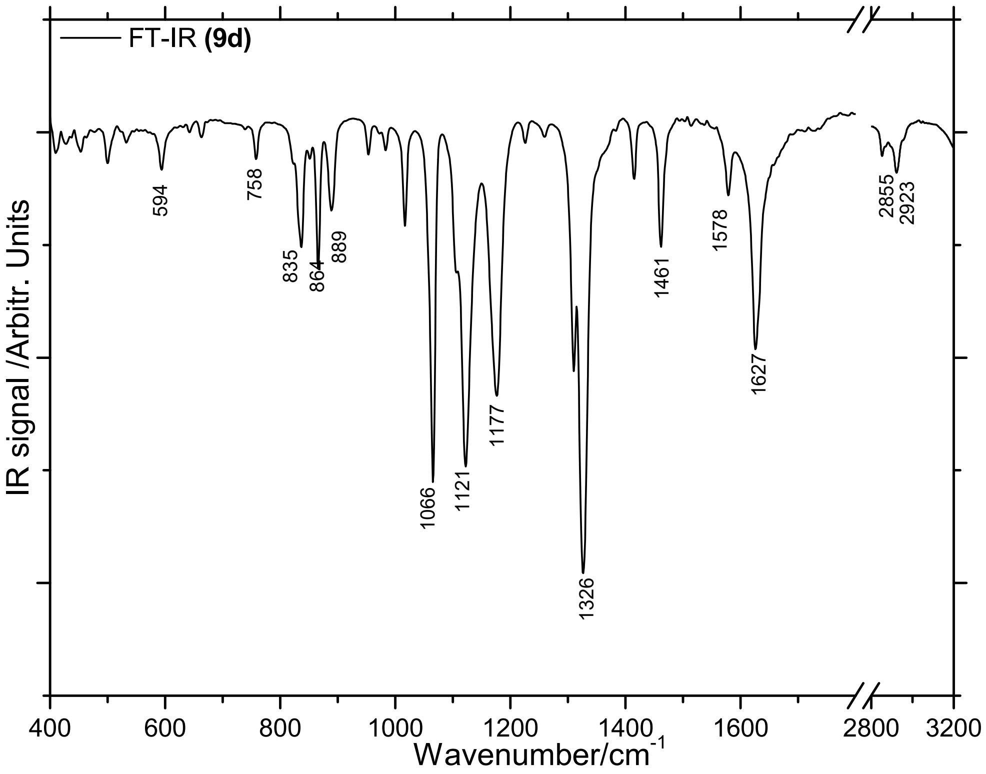

Spectra S10: FT-IR of compound **9d**

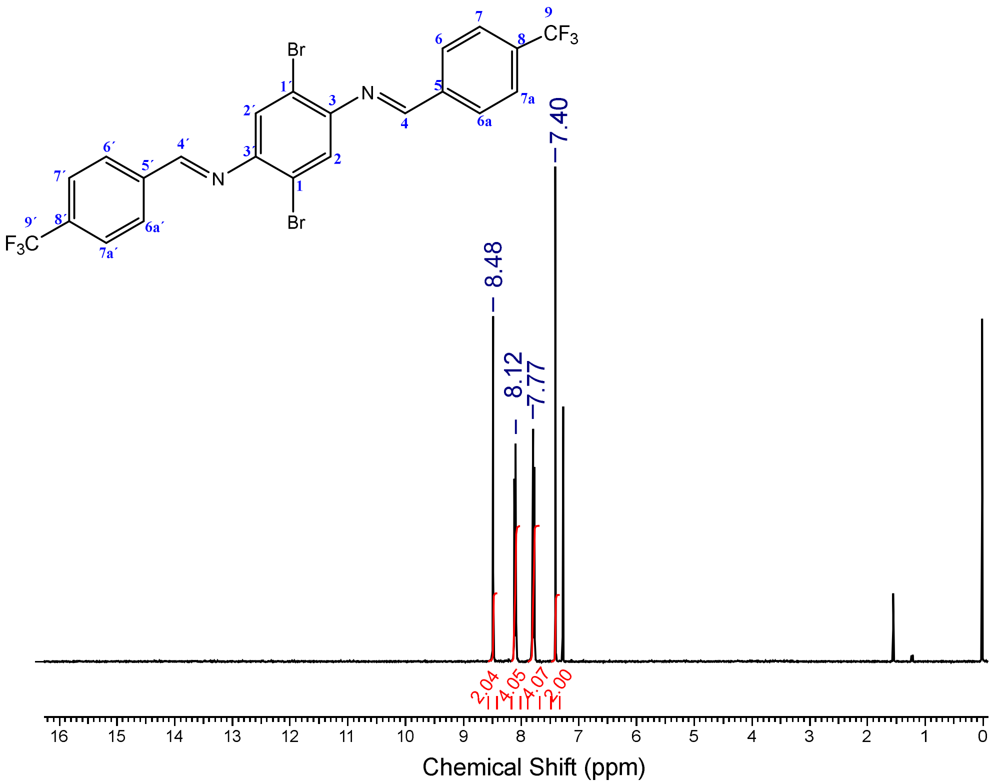

Spectra S11: ^{1}H-NMR of compound **9d**

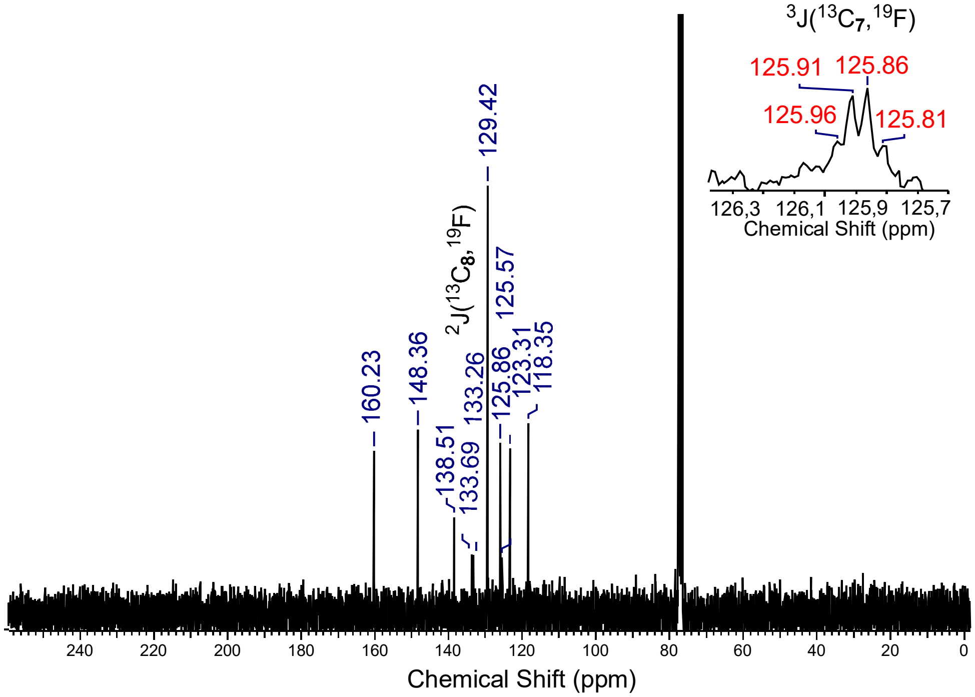

Spectra S12: ^{13}C-NMR of compound **9d**

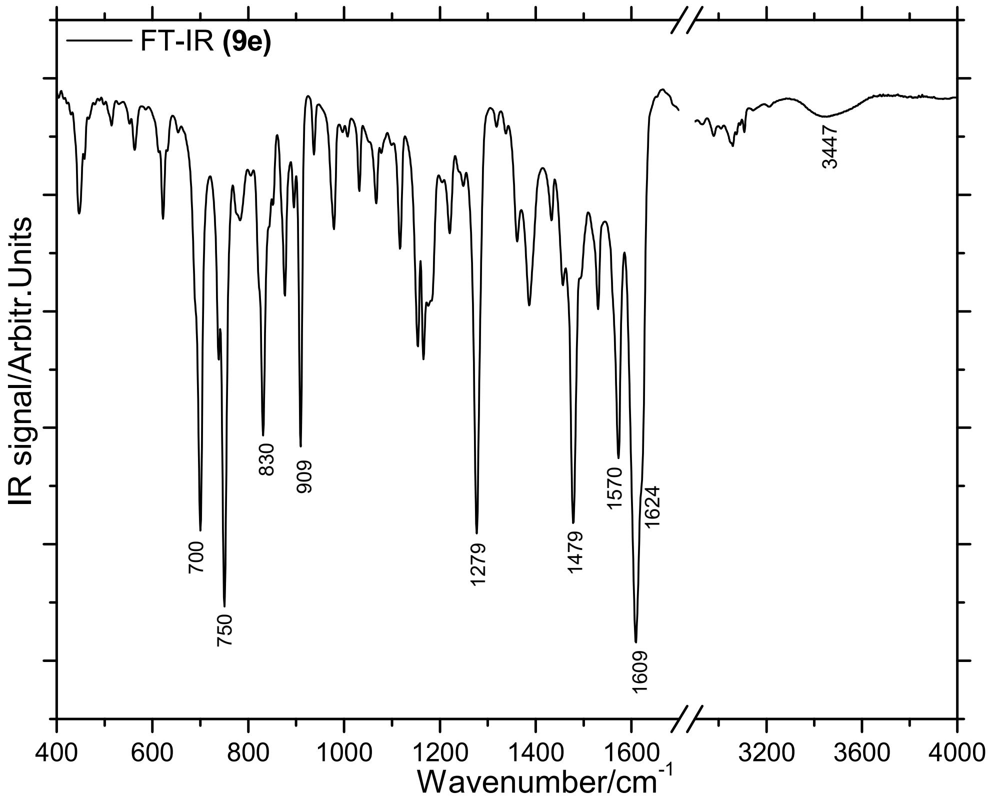

Spectra S13: FT-IR of compound **9e**

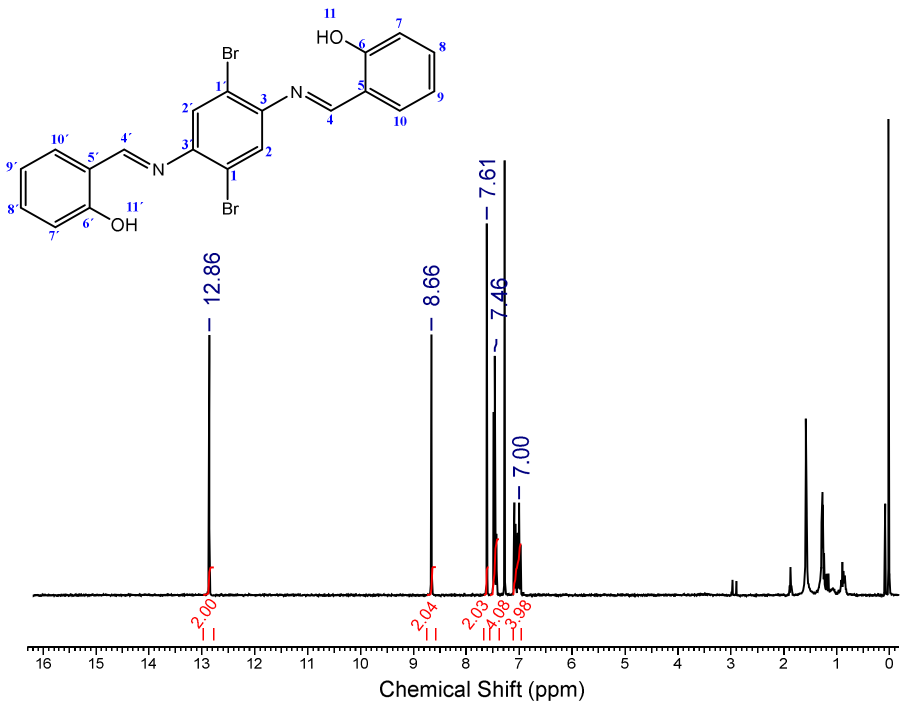

Spectra S14: ^{1}H-NMR of compound **9e**

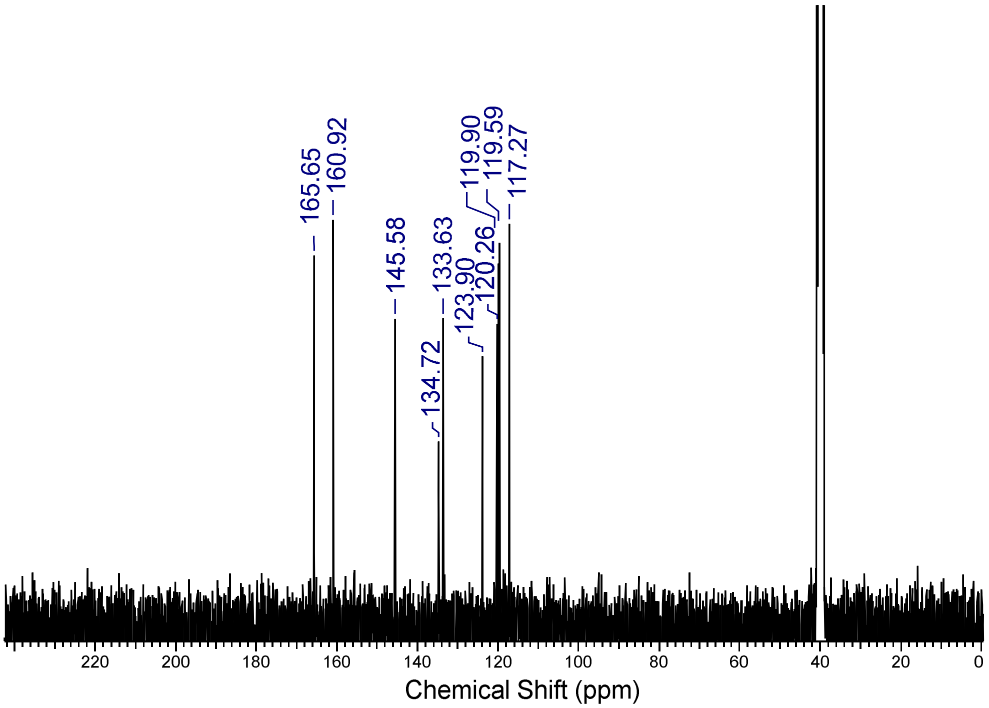

Spectra S15: ^{13}C-NMR of compound **9e**

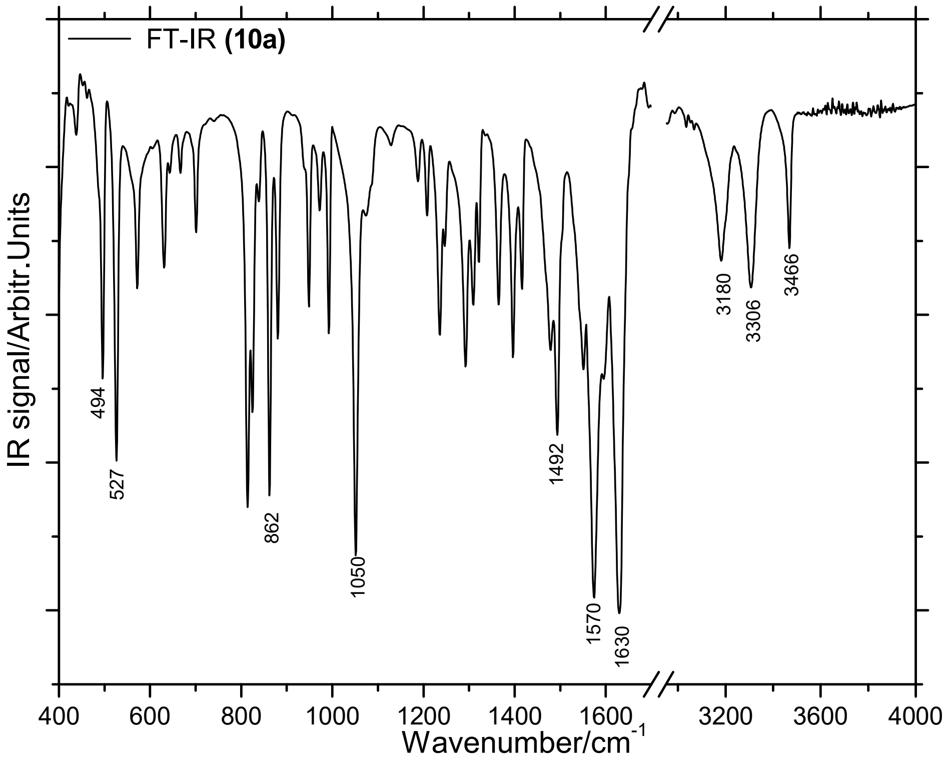

Spectra S16: FT-IR of compound **10a**

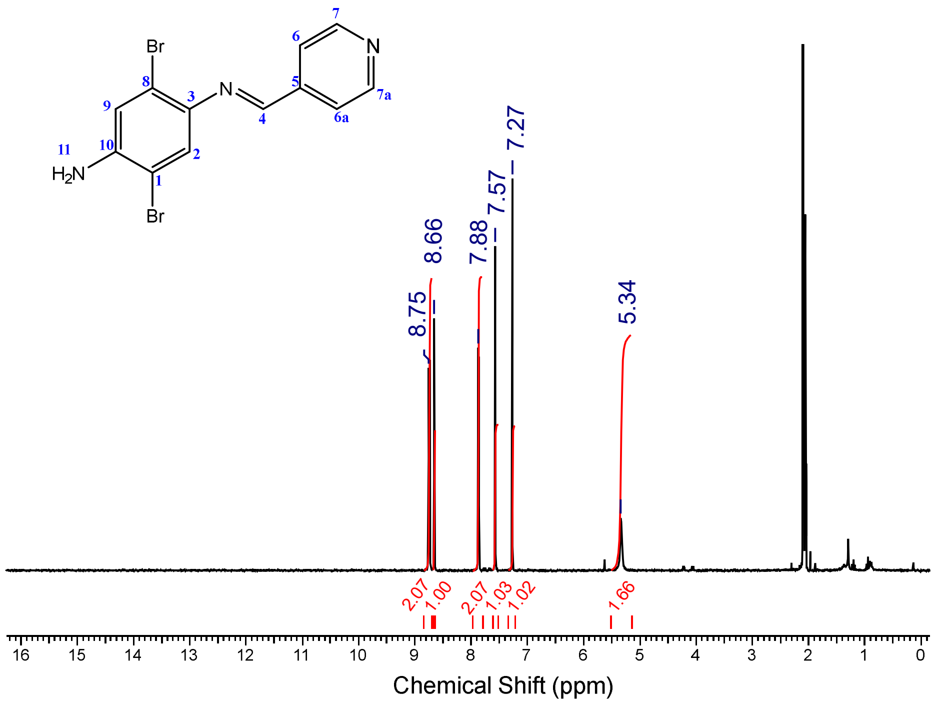

Spectra S17: ^{1}H-NMR of compound **10a**

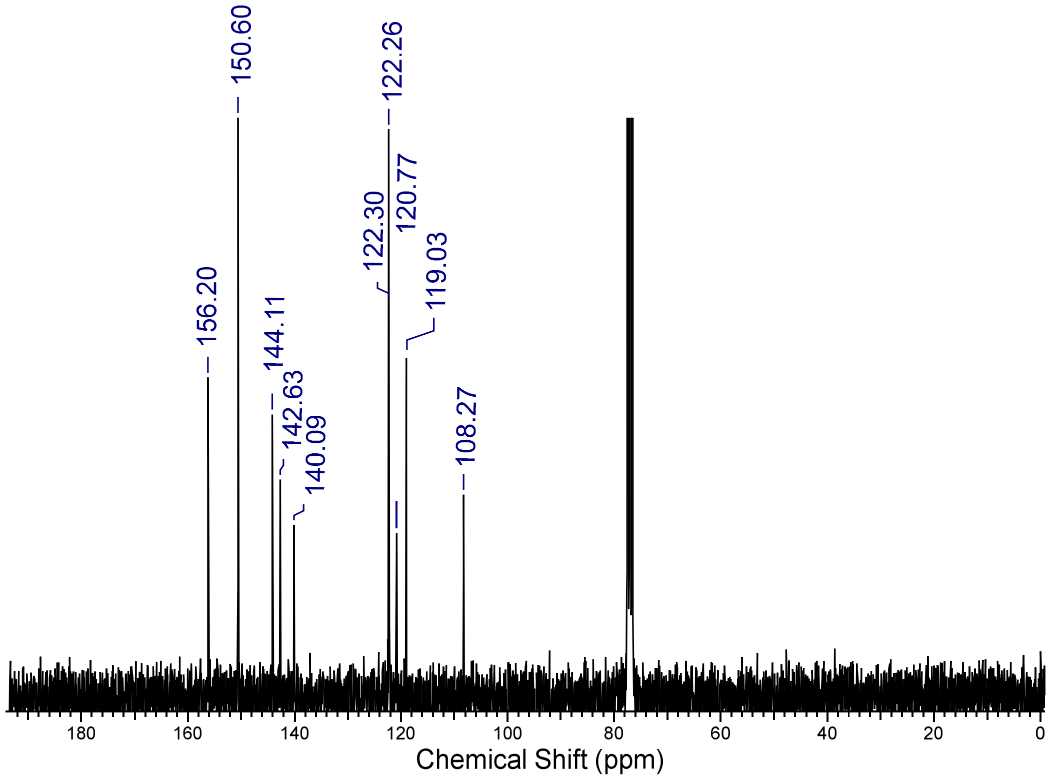

Spectra S18: ^{13}C-NMR of compound **10a**

Figures S1-S6: Molecular docking results

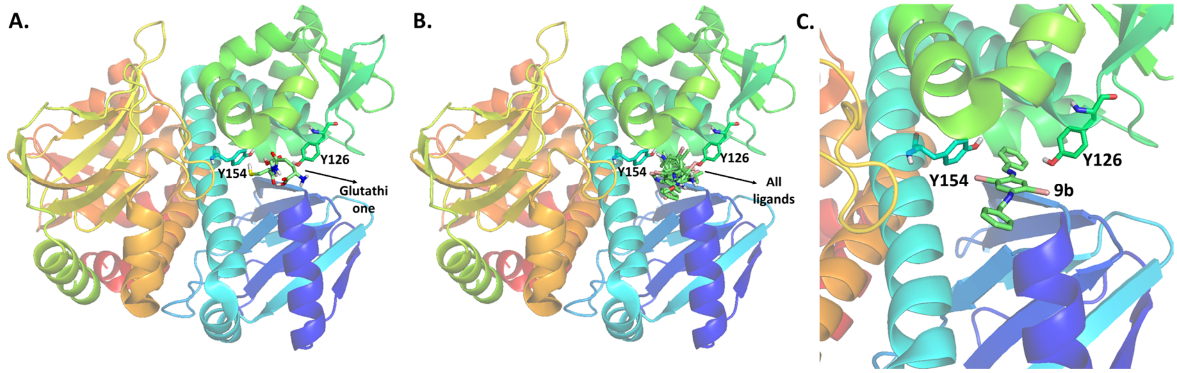

Figure S1: Molecular docking results for active compounds on positive regulatory factor A (PrfA, PDB ID: ILRR). **A.** 3D-overview for native ligand on the active site of PrfA. **B.** 3D-overview for active compounds on the active site of PrfA. **C.** Detailed interaction between key residues and the most active compound (**9b**).

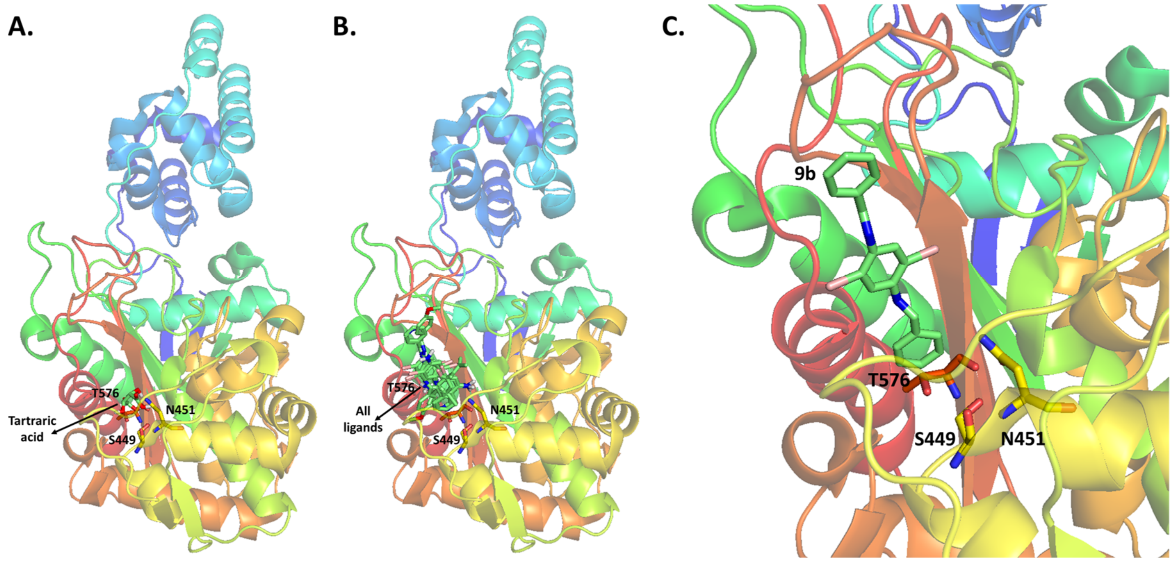

Figure S2: Molecular docking results for active compounds on penicillin-binding protein 4 (PBPs4, PDB ID: 3ZG8). **A.** 3D-overview for native ligand the on active site of PBPs4. **B.** 3D-overview for active compounds the on active site of PBPs4. **C.** Detailed interaction between key residues and the most active compound (**9b**).

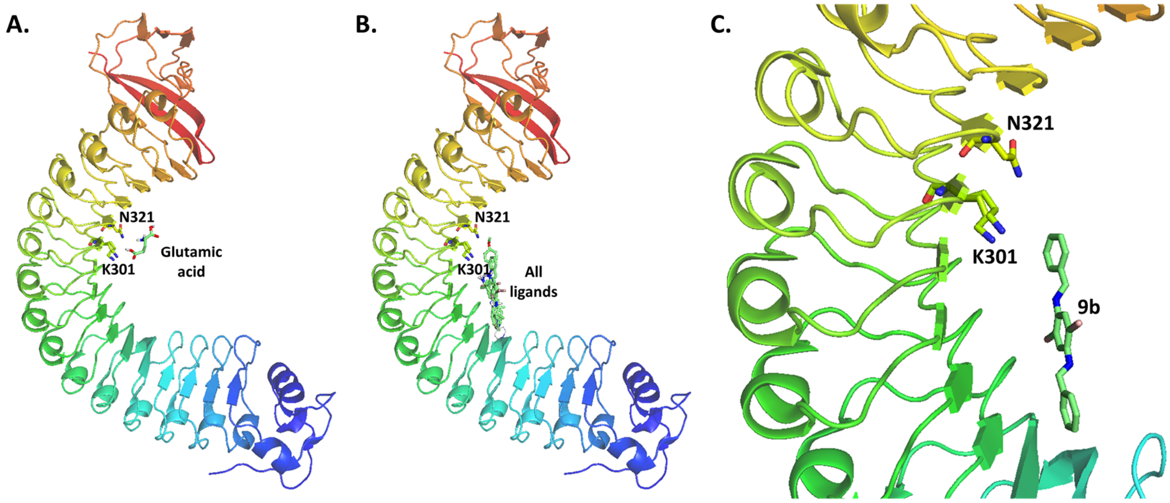

Figure S3: Molecular docking results for active compounds on internalin A (PDB ID: 1O6V). **A.** 3D-overview for native ligand on the active site of internalin A. **B.** 3D-overview for active compounds on the active site of internalin A. **C.** Detailed interactions between key residues and the most active compound (**9b**).

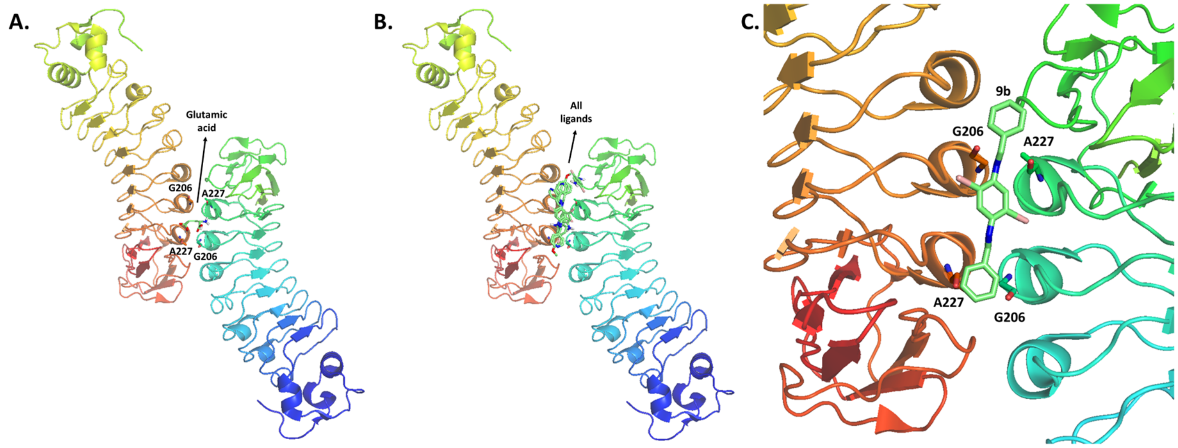

Figure S4: Molecular docking results for the active compounds against internalin B (PDB ID: 2WQU). **A.** 3D-overview for native ligand on the active site of internalin B. **B.** 3D-overview for active compounds on the active site of internalin B. **C.** Detailed interaction between key residues and the most active compound (**9b**).

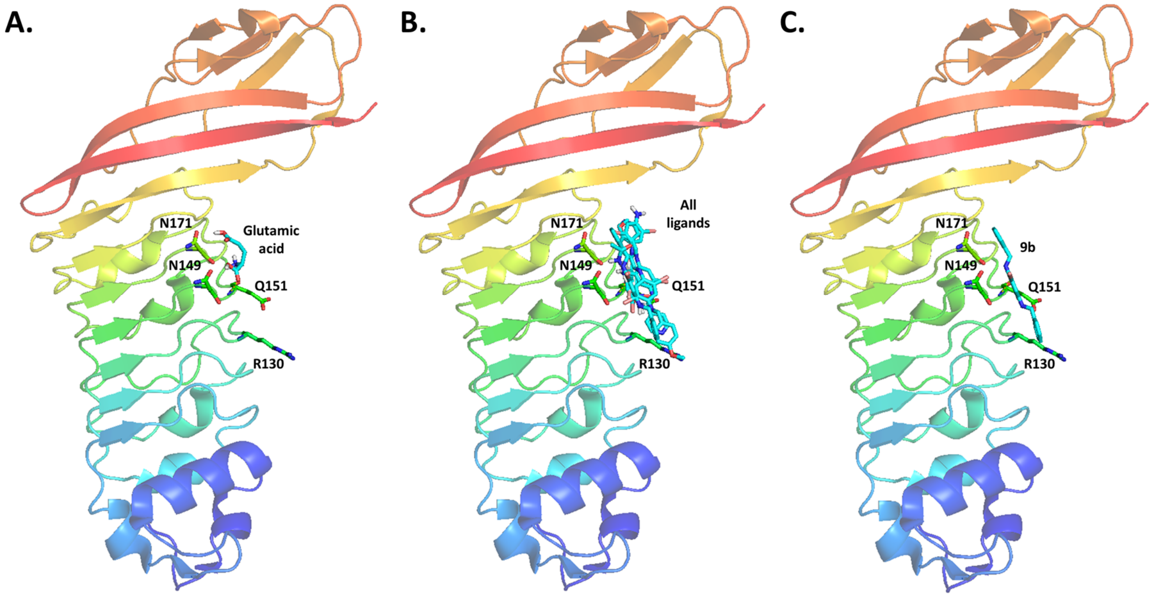

Figure S5: Molecular docking result for active compounds against internalin C (PDB ID: 1XEU). **A.** 3D-Overview for native ligand on the active site of internalin C. **B.** 3D-overview for active compounds on the active site of internalin C. **C.** Detailed interaction between key residues and the most active compound (**9b**).

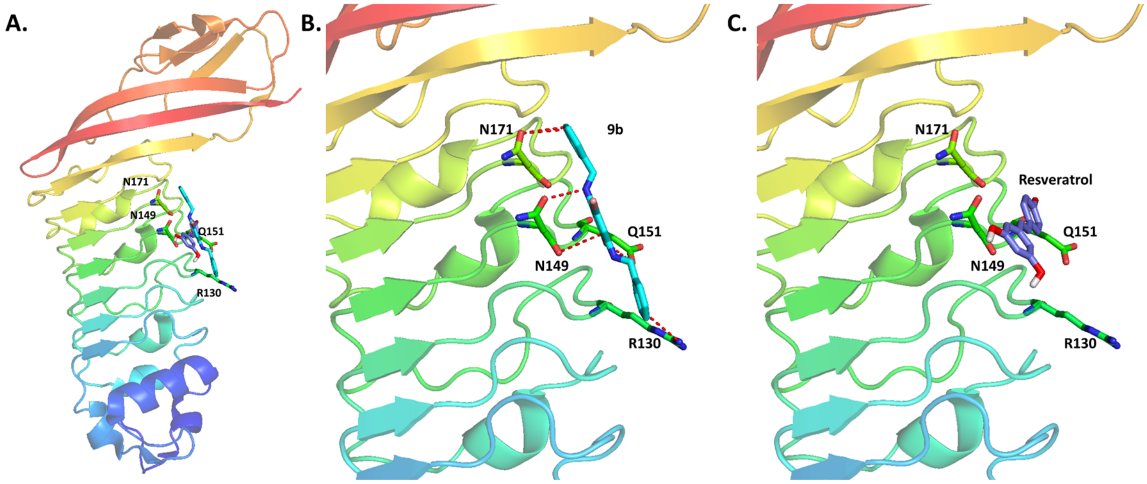

Figure S6: Molecular docking results for compound **9b** and **resveratrol** against internalin C (PDB ID: 1XEU). **A.** 3D-overview for compound **9b** and **resveratrol** on the active site of internalin C. **B.** Detailed interaction between key residues and compound **9b. C.** Detailed interaction between key residues and **resveratrol**.
